# Correction: Anatomical analysis of antebrachial cutaneous nerve distribution pattern and its clinical implications for sensory reconstruction

**DOI:** 10.1371/journal.pone.0223916

**Published:** 2019-10-10

**Authors:** The PLOS ONE Staff

Fig 4 and the Fig 4 legend are incorrect. The publisher apologizes for the errors. Please see the correct Fig 4 and Fig 4 legend here.

**Fig 4:**
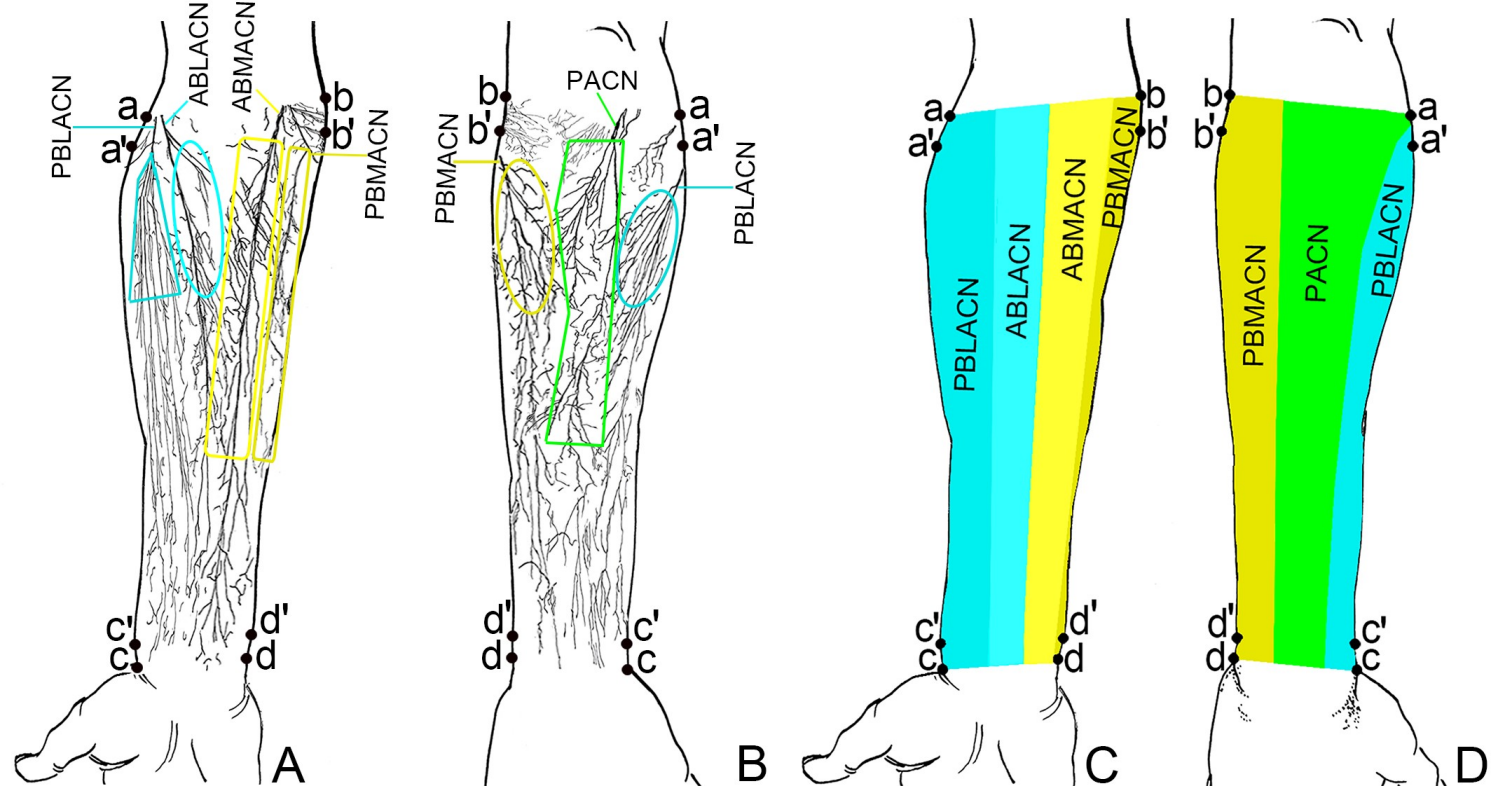
Distribution pattern and innervation area of the forearm cutaneous nerve. (A) Sketch map of cutaneous nerve distribution in the anterior forearm. The light blue and light yellow frames represent the ideal donor sites for the design of flaps with ABLACN and ABMACN, respectively. (B) Sketch map of cutaneous nerve distribution in the posterior forearm. The dark blue, dark yellow and the green frames represent the ideal donor site for the design of flaps with PBLACN, PBMACN and PACN, respectively. (C) Area innervated by cutaneous nerves of anterior forearm. (D) Area innervated by cutaneous nerves of posterior forearm.
