# Supplementary material for: Anatomical analysis of antebrachial cutaneous nerve distribution pattern and its clinical implications for sensory reconstruction
Source: PLoS One. 2019 Sep 11;14(9):e0222335. doi: 10.1371/journal.pone.0222335 (PMC6738927; doi:10.1371/journal.pone.0222335)
Supplement: Attachment — Submitted filename: Response to Reviewers.doc [file pone.0222335.s001.doc]

**Detailed Response to Reviewers**

Jul 25, 2019
Academic Editor,
PLoS One
PONE-D-19-16142
Dear Editor:

Thank you for your letter on Jul 25, 2019 regarding this manuscript. We would also like to thank the reviewers for their helpful comments. We are now submitting a revised version as detailed below that addresses these comments (original comments from reviewers and the editor are in italics). All changes have been indicated in red in the revised manuscript.

**Response to Reviewer#1:**
*The paper is original and adds to the knowledge base in this area using a novel approach to staining and defining the branching anatomy. The paper is well written and no substantial revisions are required. In line 73 the “A” should be non capital and in line 173 there should be a space between “Fig” and “3”*
**Response:**

Thank you for your positive comments. As for the two errors you pointed out, we have revised the text accordingly.

**Response to Reviewer #2:**

*1. THIS STUDY IS A VERY GOOD CADAVERIC WORK, DISPLAYING FOREARM* *SENSITIVE DISTRUBUTION WITH SOME LIMITATIONS CONCERNING RACE DIFFERENCES.*

**Response:**

(1) Thank you for your positive comment.

(2) [Because](https://www.collinsdictionary.com/dictionary/english/because) of the [restrictions](https://www.collinsdictionary.com/dictionary/english/restriction) [specimen res](https://www.collinsdictionary.com/dictionary/english/specimen)ource, Our study could not answer racial differences indeed.

(3) However, we did the following literature search about the current [understanding](https://www.collinsdictionary.com/dictionary/english/understanding) [of](https://www.collinsdictionary.com/dictionary/english/of_1) [the](https://www.collinsdictionary.com/dictionary/english/the_1) “SENSITIVE DISTRUBUTION” in [different](https://www.collinsdictionary.com/dictionary/english/different) [races](https://www.collinsdictionary.com/dictionary/english/races):

① Antebrachial Cutaneous Nerve trunk and its main branches’ distribution have been reported by authors in American, China, Austria, and Korea [1-8]. The nerve distributions described in these studies [are](https://www.collinsdictionary.com/dictionary/english/are) [essentially](https://www.collinsdictionary.com/dictionary/english/essentially) consistent. There are [variations](https://www.collinsdictionary.com/dictionary/english/variation) between [individual](https://www.collinsdictionary.com/dictionary/english/individual) samples but none [mention](https://www.collinsdictionary.com/dictionary/english/mention)ed [racial](https://www.collinsdictionary.com/dictionary/english/racial) [differences](https://www.collinsdictionary.com/dictionary/english/difference).

② Studies by Lauria et al show that there are racial differences in cutaneous innervation of the leg among European, American and Asian populations [9]. However, [Collongues et al.](https://www.ncbi.nlm.nih.gov/pubmed/?term=Collongues N%5BAuthor%5D&cauthor=true&cauthor_uid=29370274) found that no racial difference showed in clinical correlations [10].

③ Tenny et al [11] in 1984 used the lateral antebrachial cutaneous nerve graft for traumatic digital nerve defects where they found that “age” was a more important prognostic factor for sensibility. However, [The](https://www.collinsdictionary.com/dictionary/english/the_1) [study](https://www.collinsdictionary.com/dictionary/english/study) [did](https://www.collinsdictionary.com/dictionary/english/did) [not](https://www.collinsdictionary.com/dictionary/english/not_1) [mention](https://www.collinsdictionary.com/dictionary/english/mention) [racial](https://www.collinsdictionary.com/dictionary/english/racial) [differences](https://www.collinsdictionary.com/dictionary/english/difference).

④ [Our](https://www.collinsdictionary.com/dictionary/english/our_1) [study](https://www.collinsdictionary.com/dictionary/english/study) [is](https://www.collinsdictionary.com/dictionary/english/is_1) [about](https://www.collinsdictionary.com/dictionary/english/about) [the](https://www.collinsdictionary.com/dictionary/english/the_1) [overall](https://www.collinsdictionary.com/dictionary/english/overall) [display](https://www.collinsdictionary.com/dictionary/english/display) [and](https://www.collinsdictionary.com/dictionary/english/and_1) [comparison](https://www.collinsdictionary.com/dictionary/english/comparison) [of](https://www.collinsdictionary.com/dictionary/english/of_1) [the](https://www.collinsdictionary.com/dictionary/english/the_1) forearm [nerve](https://www.collinsdictionary.com/dictionary/english/neural) [distribution](https://www.collinsdictionary.com/dictionary/english/distribution) [density](https://www.collinsdictionary.com/dictionary/english/density) [in](https://www.collinsdictionary.com/dictionary/english/in_1) each region. [As](https://www.collinsdictionary.com/dictionary/english/as_1) [far](https://www.collinsdictionary.com/dictionary/english/far) [as](https://www.collinsdictionary.com/dictionary/english/as_1) [we](https://www.collinsdictionary.com/dictionary/english/we) [know](https://www.collinsdictionary.com/dictionary/english/know), neurosensitivity [cannot](https://www.collinsdictionary.com/dictionary/english/cannot) [be](https://www.collinsdictionary.com/dictionary/english/be_1) [equated](https://www.collinsdictionary.com/dictionary/english/equate) [with](https://www.collinsdictionary.com/dictionary/english/with_1) [nerve](https://www.collinsdictionary.com/dictionary/english/nerve) [density](https://www.collinsdictionary.com/dictionary/english/density), [and](https://www.collinsdictionary.com/dictionary/english/and_1) [sensitivity](https://www.collinsdictionary.com/dictionary/english/sensitivity) [may](https://www.collinsdictionary.com/dictionary/english/may) [be](https://www.collinsdictionary.com/dictionary/english/be_1) [affected](https://www.collinsdictionary.com/dictionary/english/affected) [by](https://www.collinsdictionary.com/dictionary/english/by_1) [gender](https://www.collinsdictionary.com/dictionary/english/gender), [age](https://www.collinsdictionary.com/dictionary/english/age_1), [personal](https://www.collinsdictionary.com/dictionary/english/personal) [living](https://www.collinsdictionary.com/dictionary/english/living) [conditions](https://www.collinsdictionary.com/dictionary/english/conditions), [etc](https://www.collinsdictionary.com/dictionary/english/etc).

[In](https://www.collinsdictionary.com/dictionary/english/overall) general, [we](https://www.collinsdictionary.com/dictionary/english/we) [hope](https://www.collinsdictionary.com/dictionary/english/hope) [that](https://www.collinsdictionary.com/dictionary/english/that) [further](https://www.collinsdictionary.com/dictionary/english/further) research in the future [will](https://www.collinsdictionary.com/dictionary/english/will) [confirm](https://www.collinsdictionary.com/dictionary/english/confirm) [whether](https://www.collinsdictionary.com/dictionary/english/whether) [there](https://www.collinsdictionary.com/dictionary/english/there_1) [are](https://www.collinsdictionary.com/dictionary/english/are) [racial](https://www.collinsdictionary.com/dictionary/english/racial) [differences](https://www.collinsdictionary.com/dictionary/english/difference) [in](https://www.collinsdictionary.com/dictionary/english/in_1) [the](https://www.collinsdictionary.com/dictionary/english/the_1) forearm [nerve](https://www.collinsdictionary.com/dictionary/english/neural) [distribution](https://www.collinsdictionary.com/dictionary/english/distribution).

1. Finneran JJ, [Sandhu N](https://www.ncbi.nlm.nih.gov/pubmed/?term=Sandhu N%5BAuthor%5D&cauthor=true&cauthor_uid=29732596). Ultrasound-Guided Posterior Antebrachial Cutaneous Nerve Block: Technical Description and Block Distribution in Healthy Volunteers. [J Ultrasound Med.](https://www.ncbi.nlm.nih.gov/pubmed/29732596) 2019; 38: 239-242. doi: 10.1002/jum.14678. PMID: 29732596.

2. Singhal S, Rao VV, Ravindranath R. Variations in brachial plexus and the relationship of median nerve with the axillary artery: A case report. J Brachial Plex Peripher Nerve Inj. 2007; 2: 21. doi: [10.1186/1749-7221-2-21](https://doi.org/10.1186/1749-7221-2-21). PMID: 17915015.

3. Race CM, Saldana MJ. Anatomic course of the medial cutaneous nerves of the arm. J Hand Surg Am. 1991; 16: 48–52. PMID: 1995693.

4. [Zhang FH](https://www.ncbi.nlm.nih.gov/pubmed/?term=Zhang FH%5BAuthor%5D&cauthor=true&cauthor_uid=16783807), [Topp SG](https://www.ncbi.nlm.nih.gov/pubmed/?term=Topp SG%5BAuthor%5D&cauthor=true&cauthor_uid=16783807), [Zhang WJ](https://www.ncbi.nlm.nih.gov/pubmed/?term=Zhang WJ%5BAuthor%5D&cauthor=true&cauthor_uid=16783807), [Zheng HP](https://www.ncbi.nlm.nih.gov/pubmed/?term=Zheng HP%5BAuthor%5D&cauthor=true&cauthor_uid=16783807), [Zhang F](https://www.ncbi.nlm.nih.gov/pubmed/?term=Zhang F%5BAuthor%5D&cauthor=true&cauthor_uid=16783807). Anatomic study of distally based pedicle compound flaps with nutrient vessels of the cutaneousnerves and superficial veins of the forearm. [Microsurgery.](https://www.ncbi.nlm.nih.gov/pubmed/?term=ANATOMIC+STUDY+OF+DISTALLY+BASED+PEDICLE+COMPOUND+FLAPS+WITH+NUTRIENT+VESSELS+OF+THE+CUTANEOUS+NERVES+AND+SUPERFICIAL+VEINS+OF+THE+FOREARM) 2006; 26: 373-85. doi: [10.1002/micr.20255](https://doi.org/10.1002/micr.20255). PMID: 16783807.

5. [Benedikt S](https://www.ncbi.nlm.nih.gov/pubmed/?term=Benedikt S%5BAuthor%5D&cauthor=true&cauthor_uid=28756975), [Parvizi D](https://www.ncbi.nlm.nih.gov/pubmed/?term=Parvizi D%5BAuthor%5D&cauthor=true&cauthor_uid=28756975), [Feigl G](https://www.ncbi.nlm.nih.gov/pubmed/?term=Feigl G%5BAuthor%5D&cauthor=true&cauthor_uid=28756975), [Koch H](https://www.ncbi.nlm.nih.gov/pubmed/?term=Koch H%5BAuthor%5D&cauthor=true&cauthor_uid=28756975). Anatomy of the medial antebrachial cutaneous nerve and its significance in ulnar nerve surgery: An anatomical study. [J Plast Reconstr Aesthet Surg.](https://www.ncbi.nlm.nih.gov/pubmed/?term=The+Anatomy+of+the+Medial+Antebrachial+Cutaneous+Nerve+and+its+Significance+in+Ulnar+Nerve+Surgery+–+an+Anatomical+Study) 2017 Nov; 70: 1582-1588. doi: 10.1016/j.bjps.2017.06.025. PMID: 28756975.

6. Moritz T, Prosch H, Pivec CH, et al. High-resolution ultrasound visualization of the subcutaneous nerves of the forearm: A feasibility study in anatomic specimens. Muscle Nerve. 2014; 49: 676-679. doi: 10.1002/mus.24064. PMID: 24038104.

7. [Im HS](https://www.ncbi.nlm.nih.gov/pubmed/?term=Im HS%5BAuthor%5D&cauthor=true&cauthor_uid=28758079), [Im JY](https://www.ncbi.nlm.nih.gov/pubmed/?term=Im JY%5BAuthor%5D&cauthor=true&cauthor_uid=28758079), [Kim KH](https://www.ncbi.nlm.nih.gov/pubmed/?term=Kim KH%5BAuthor%5D&cauthor=true&cauthor_uid=28758079),[Kim DH](https://www.ncbi.nlm.nih.gov/pubmed/?term=Kim DH%5BAuthor%5D&cauthor=true&cauthor_uid=28758079), [Park BK](https://www.ncbi.nlm.nih.gov/pubmed/?term=Park BK%5BAuthor%5D&cauthor=true&cauthor_uid=28758079). Ultrasonographic Study of the Anatomical Relationship Between the Lateral Antebrachial Cutaneous Nerve and the Cephalic Vein. [Ann Rehabil Med.](https://www.ncbi.nlm.nih.gov/pubmed/?term=Ultrasonographic+Study+of+the+Anatomical+Relationship+Between+the+Lateral+Antebrachial+Cutaneous+Nerve+and+the+Cephalic+Vein) 2017; 41: 421-425. doi: 10.5535/arm.2017.41.3.421. PMID: 28758079.

8. Oh CH, Park NS, Kim JM, Kim MW. Determination of an ideal stimulation site of the medial antebrachial cutaneous nerve using ultrasound and investigation of the efficiency. Ann Rehabil Med. 2014; 38: 836–842. doi: 10.5535/arm.2014.38.6.836. PMID: 25566484.

9. Lauria G, Bakkers M, Schmitz C, Lombardi R, Penza P, Devigili G, et al. Intraepidermal nerve fiber density at the distal leg: a worldwide normative reference study. J Peripher Nerv Syst. 2010; 15: 202–207. doi: 10.1111/j.1529-8027.2010.00271.x. PMID: 21040142.

10. [Collongues N](https://www.ncbi.nlm.nih.gov/pubmed/?term=Collongues N%5BAuthor%5D&cauthor=true&cauthor_uid=29370274), [Samama B](https://www.ncbi.nlm.nih.gov/pubmed/?term=Samama B%5BAuthor%5D&cauthor=true&cauthor_uid=29370274), [Schmidt-Mutter C](https://www.ncbi.nlm.nih.gov/pubmed/?term=Schmidt-Mutter C%5BAuthor%5D&cauthor=true&cauthor_uid=29370274), [Chamard-Witkowski L](https://www.ncbi.nlm.nih.gov/pubmed/?term=Chamard-Witkowski L%5BAuthor%5D&cauthor=true&cauthor_uid=29370274), [Debouverie M](https://www.ncbi.nlm.nih.gov/pubmed/?term=Debouverie M%5BAuthor%5D&cauthor=true&cauthor_uid=29370274), [Chanson JB](https://www.ncbi.nlm.nih.gov/pubmed/?term=Chanson JB%5BAuthor%5D&cauthor=true&cauthor_uid=29370274). et al. Quantitative and qualitative normativedataset for intraepidermal nerve fibers usingskin biopsy. PLOS ONE. 2018; 13: e0191614. doi: 10.1371/journal.pone.0191614. PMID: 29370274.

11. [Tenny JR](https://www.ncbi.nlm.nih.gov/pubmed/?term=Tenny JR%5BAuthor%5D&cauthor=true&cauthor_uid=6501333), [Lewis RC](https://www.ncbi.nlm.nih.gov/pubmed/?term=Lewis RC%5BAuthor%5D&cauthor=true&cauthor_uid=6501333). Digital nerve-grafting for traumatic defects. Use of the lateral antebrachial cutaneous nerve.JBone Joint Surg Am. 1984; 66: 1375–1379. PMID: 6501333.

*2. CLINICAL USE OF THIS KNOWLEDGE ( YOUR FINDINGS ) IS DEBATABLE SINCE, FLAP ELEVATION STILL DEPENDS ON VASCULAR (PERFORATOR VESSEL) DISTRIBUTION.*

*I HAVE SOME DOUBTS REGARDING DONOR AREA SENSITIVE NERVE THICKNESS IS DIRECTLY CORRELATED WITH BETTER SENSITIVE OUTCOME, YOU WOULD RATHER SMOOTHEN YOUR IDEAS OR SUPPORT WTH MORE DATA.*

**Response:**

[We](https://www.collinsdictionary.com/dictionary/english/we) [completely](https://www.collinsdictionary.com/dictionary/english/complete) [agree](https://www.collinsdictionary.com/dictionary/english/agree) [with](https://www.collinsdictionary.com/dictionary/english/with_1) [you](https://www.collinsdictionary.com/dictionary/english/you): “FLAP ELEVATION STILL DEPENDS ON VASCULAR (PERFORATOR VESSEL) DISTRIBUTION”. Our study aimed at [improv](https://www.collinsdictionary.com/dictionary/english/improve)ing [the](https://www.collinsdictionary.com/dictionary/english/the_1) [quality](https://www.collinsdictionary.com/dictionary/english/excellent-quality) [of](https://www.collinsdictionary.com/dictionary/english/of_1) [the](https://www.collinsdictionary.com/dictionary/english/the_1) [flaps](https://www.collinsdictionary.com/dictionary/english/flap) by considering the recipient [sensory](https://www.collinsdictionary.com/dictionary/english/sensory) recovery so that [the](https://www.collinsdictionary.com/dictionary/english/the_1) [clinicians](https://www.collinsdictionary.com/dictionary/english/clinician) can use this knowledge to [guide](https://www.collinsdictionary.com/dictionary/english/guide) [the](https://www.collinsdictionary.com/dictionary/english/the_1) [design](https://www.collinsdictionary.com/dictionary/english/design) [of](https://www.collinsdictionary.com/dictionary/english/of_1) [the](https://www.collinsdictionary.com/dictionary/english/the_1) [flaps](https://www.collinsdictionary.com/dictionary/english/flap). We have also expressed [these](https://www.collinsdictionary.com/dictionary/english/these) [views](https://www.collinsdictionary.com/dictionary/english/view) [in](https://www.collinsdictionary.com/dictionary/english/in_1) " Introduction, Paragraph 2 ".

The [theoretical](https://www.collinsdictionary.com/dictionary/english/theoretical) [design](https://www.collinsdictionary.com/dictionary/english/design) of this study is based on references 28-30 listed in this paper. [Please](https://www.collinsdictionary.com/dictionary/english/as_1) also refer to the “[Discussion](https://www.collinsdictionary.com/dictionary/english/discuss) section, [paragraph](https://www.collinsdictionary.com/dictionary/english/paragraph) 5”. “Sensory reconstruction primarily occurs as follows: the reception area and surrounding nerve fibers grow into the flap through the scar tissue (peripheral manner), or the recipient nerves are anastomosed with the main innervating nerve of the flap (central manner). The direction of nerve regeneration can be guided by the alignment of nerve tissues, so that Schwann cells at both ends can directly restore material communication channels, and the regenerated nerve fibers can grow smoothly into the distal neurotubules [28]. However, the important factors for flap recovery are distribution of sensory nerves in the donor site of the flap, the choice of the cutaneous nerve, and its anastomotic site [29, 30]. Therefore, **if the recipient nerve is rich, a rich donor nerve should be matched to increase the probability of nerve contact.**” [We](https://www.collinsdictionary.com/dictionary/english/we) [hope](https://www.collinsdictionary.com/dictionary/english/hope) [that](https://www.collinsdictionary.com/dictionary/english/that) this reviewer [will](https://www.collinsdictionary.com/dictionary/english/will) [accept](https://www.collinsdictionary.com/dictionary/english/accept) [our](https://www.collinsdictionary.com/dictionary/english/these) [views](https://www.collinsdictionary.com/dictionary/english/view).

28. Ide C, Tohyama K, Yokota R, Nitatori T, Onodera S. Schwann cell basal lamina and nerve regeneration. Brain Res. 1983; 288: 61-75. doi: [10.1016/0006-8993(83)90081-1](https://doi.org/10.1016/0006-8993(83)90081-1). PMID: [6661636](https://www.ncbi.nlm.nih.gov/pubmed/6661636).

29. Feng SM, Wang AG, Zhang ZY, [Sun QQ](https://www.ncbi.nlm.nih.gov/pubmed/?term=Sun QQ%5BAuthor%5D&cauthor=true&cauthor_uid=28925493), [Tao YL](https://www.ncbi.nlm.nih.gov/pubmed/?term=Tao YL%5BAuthor%5D&cauthor=true&cauthor_uid=28925493), [Zhou MM](https://www.ncbi.nlm.nih.gov/pubmed/?term=Zhou MM%5BAuthor%5D&cauthor=true&cauthor_uid=28925493), [et](https://www.ncbi.nlm.nih.gov/pubmed/?term=Hao YJ%5BAuthor%5D&cauthor=true&cauthor_uid=28925493) al. Repair and sensory reconstruction of the children's finger pulp defects with perforator pedicled propeller flap in proper digital artery. Eur Rev Med Pharmacol Sci. 2017; 21: 3533-3537. PMID: [28925493](https://www.ncbi.nlm.nih.gov/pubmed/28925493).

30. Zhu L, Zhang J, Song X, [Hou W](https://www.ncbi.nlm.nih.gov/pubmed/?term=Hou W%5BAuthor%5D&cauthor=true&cauthor_uid=28370156), [Wu S](https://www.ncbi.nlm.nih.gov/pubmed/?term=Wu S%5BAuthor%5D&cauthor=true&cauthor_uid=28370156), [Chen W](https://www.ncbi.nlm.nih.gov/pubmed/?term=Chen W%5BAuthor%5D&cauthor=true&cauthor_uid=28370156), et al. Sensory recovery of non-innervated free flaps and nasolabial island flaps used for tongue reconstruction of oncological defects. J Oral Rehabil. 2017; 44: 736-748. doi: [10.1111/joor.12510](https://doi.org/10.1111/joor.12510). PMID: [28370156](https://www.ncbi.nlm.nih.gov/pubmed/28370156).

**Response to Reviewer #3:**

*1. Advantages：omit.*

*2. Disadvantages:
- the process of pictures conversion and further image processing is not well described.
- although authors describe the results thickness, area and nerve branch density of each region (p.10) they provide only general ANOVA result (p. 11) without post-hoc analysis
- clinical implications are hard to drawn due to big amount of data in multiple regions - most suitable donor sites could be marked on separate figure.*

**Response:**

In order to better intuitively understand the ideal donor sites, we have marked in Figs 4A and 4B. We did not add new figures for the concern of reducing the pages of the manuscript.

About the clinical significance of this study: We analyzed the clinical application of three cutaneous nerves in the forearm based on the experimental results. These descriptions are in the fourth paragraph of the discussion section.

We added post-hoc analysis in statistical processing (see revised text).

**Response to Editor**

Thank you for your consideration of this revision. The editing of the text is based on the template of your journal.

Sincerely,

Shengbo Yang
Department of Anatomy
Zunyi Medical University
