# Supplementary material for: Anatomical analysis of antebrachial cutaneous nerve distribution pattern and its clinical implications for sensory reconstruction
Source: PLoS One. 2019 Sep 11;14(9):e0222335. doi: 10.1371/journal.pone.0222335 (PMC6738927; doi:10.1371/journal.pone.0222335)
Supplement: Attachment — Submitted filename: Response to Reviewers.doc [file pone.0222335.s002.doc]

**Detailed Response to Reviewers**

Aug 25, 2019
Academic Editor,
PLOS ONE
PONE-D-19-16142R1

Dear Editor:

Thank you for your letter on Aug 23, 2019 regarding this manuscript. We would also like to thank the reviewers for their helpful comments. We are now submitting a revised version as detailed below that addresses these comments (original comments from reviewers and the editor are in italics). All changes have been indicated in red in the revised manuscript.

**Response to Reviewer#2:**
*Forearm sensitive nerve distrubution is important in terms of donor area selection and flap choices so I think your manuscript will be usefull for the scientists dealing with this subject. However terminology is important as well so all "anastomosis" words must be replaced with coaptation or neuroraphy.*
**Response:**

Thank you for your positive comment. According to your comments, we have replaced "anastomosis" with coaptation.

**Response to Editor:**

We have revised the text in accordance with the comments of the reviewers.

Sincerely,

Shengbo Yang
Department of Anatomy
Zunyi Medical University
